# Decoding IL-1 receptor 1 and 2 expression profiles across organs in sepsis

**DOI:** 10.3389/fcell.2025.1675870

**Published:** 2025-11-10

**Authors:** Chuyi Tan, Weiqin Wang, Han Ma, Huan Chen, Huali Zhang, Yue Peng, Yiying Yang

**Affiliations:** 1 Key Laboratory of Sepsis Translational Medicine of Hunan, Department of Pathophysiology, Xiangya School of Basic Medicine Science, Central South University, Changsha, Hunan, China; 2 National Medicine Functional Experimental Teaching Center, Central South University, Changsha, Hunan, China; 3 Department of Critical Care Medicine, The Third Xiangya Hospital, Central South University, Changsha, Hunan, China; 4 Postdoctoral Research Station of Biology, Xiangya School of Basic Medicine Science, Central South University, Changsha, Hunan, China

**Keywords:** sepsis, IL-1, IL-1R2, IL-1R1, macrophage, neutrophil, resident macrophage, inflammation

## Abstract

**Introduction:**

Interleukin-1 (IL-1), a key inflammatory mediator, plays a critical role in the pathogenesis of sepsis. IL-1 signals through two major receptors, the signaling receptor IL-1R1 and the decoy receptor IL-1R2. However, the cell-type-specific and organ-specific expression dynamics of these receptors during sepsis remain poorly characterized.

**Methods:**

Using publicly available single-cell RNA sequencing (scRNA-seq) datasets and flow cytometry validation, we systematically analyzed the expression profiles of IL-1R1 and IL-1R2 across multiple organs—including the lung, liver, heart, and small intestine in murine models of cecal ligation and puncture (CLP)-induced sepsis.

**Results:**

We found that IL-1R1 was predominantly expressed on non-immune cells (lung fibroblasts, liver endothelial cells and heart fibroblasts), and showed increased changes during sepsis. In contrast, IL-1R2 was primarily expressed on neutrophils and monocyte-derived macrophages in healthy conditions, with minimal expression on tissue resident macrophages such as alveolar macrophages and Kupffer cells). Sepsis induced a significant upregulation of IL-1R2 on neutrophils and monocyte-derived macrophages across all organs. However, resident macrophages in the lung, liver and heart maintain low expression during sepsis.

**Discussion:**

We reveal distinct and compartmentalized expression landscapes for IL-1R1 and IL-1R2 across organs during sepsis. These findings offer a deep understanding of IL-1 receptors biology and shed light on their contributions to immune modulation and tissue-specific responses in sepsis.

## 1 Introduction

Sepsis, a major global health challenge, is characterized by a dysregulated host response to infection that leads to organ dysfunction and high mortality rates (Seymour et al., 2016; Rudd et al., 2020). It is often characterized by an imbalance between pro-inflammatory and anti-inflammatory responses within the host immune system, leading to immune system overactivation and excessive production of cytokines, a phenomenon referred to as a “cytokine storm” (Tisoncik et al., 2012). Interleukin-1 (IL-1) plays a critical role in both the initiation and amplification of the inflammation response, and is also an essential component of host defense against infection during sepsis (Dinarello, 2011; Weber et al., 2010).

IL-1 exerts its biological effects by binding to members of the IL-1 receptor (IL-1R) family, a group of structurally related transmembrane proteins that play diverse roles in regulating the IL-1 mediated immune response (Boraschi and Tagliabue, 2013; O'Neill, 2008). The family includes the primary signaling receptor, IL-1R1, and the decoy receptor, IL-1R2. Upon binding of IL-1α or IL-1β, IL-1R1 forms a heterodimeric complex with the IL-1 receptor accessory protein (IL-1RAcP) (Boraschi and Tagliabue, 2013; Colotta et al., 1993). The recruitment of IL-1RAcP to the IL-1/IL-1R1 complex initiates a signaling cascade involving the adaptor protein MyD88, leading to the activation of downstream kinases and ultimately the activation of transcription factors such as NF-κB, resulting in the expression of numerous pro-inflammatory genes (O'Neill, 2008; Colotta et al., 1993; Mantovani et al., 2019).

In contrast to IL-1R1, IL-1R2 acts as a decoy receptor. IL-1R2 also binds IL-1α and IL-1β with high affinity, but it lacks the intracellular Toll/IL-1 receptor (TIR) domain necessary for signal transduction (Colotta et al., 1993; Mantovani et al., 2001). Consequently, IL-1R2 effectively competes with IL-1R1 for binding IL-1, preventing it from activating the signaling pathway (Mantovani et al., 2001; Dinarello, 2018). Furthermore, soluble forms of IL-1R2 (sIL-1R2), produced by proteolytic cleavage of the membrane-bound form or by alternative splicing, can circulate and neutralize IL-1 in the extracellular space (Zhang et al., 2024; Orlando et al., 1997). Beyond IL-1R1 and IL-1R2, other members of the IL-1R family exist, including SIGIRR (Single Ig IL-1-related receptor, also known as IL-1R8), which is thought to act as a negative regulator of IL-1 and Toll-like receptor signaling (Wald et al., 2003; Sham et al., 2013). These different receptor subtypes finely tuned regulation of the IL-1 pathway, playing a critical role in balancing pro- and anti-inflammatory processes in sepsis.

Given its important role in regulating inflammation, IL-1R has been investigated as a potential therapeutic target in various inflammatory conditions, including sepsis (Gabay et al., 2010; Shakoory et al., 2016). Despite the pivotal role of IL-1 in modulating the immune system, multi-center randomized controlled trials targeting IL-1 inhibition in sepsis have yielded limited success (Zhang and Ning, 2021). This underscores the need for a further understanding of the precise regulation of IL-1 and IL-1R, which holds significant potential for developing more effective treatments for inflammatory and infectious diseases. However, a comprehensive analysis of its cell-type-specific and organ-specific dynamics of IL-1R expression during sepsis remains poorly understood.

To investigate the roles of IL-1R subtypes, specifically IL-1R1 and IL-1R2, in sepsis, we utilized single-cell RNA sequencing (scRNA-seq) and flow cytometry to comprehensively map their expression profiles across various immune cell populations and organs. This approach enabled the identification of distinct receptor dynamics, shedding light on their contributions to immune modulation and tissue-specific responses in sepsis. These findings offer a deeper understanding of IL-1 receptor biology and highlight potential therapeutic targets for managing sepsis.

## 2 Methods

### 2.1 Assessment of IL-1R1 and IL-1R2 expression in different cells of murine lung by scRNA-seq data

**FIGURE 1:**
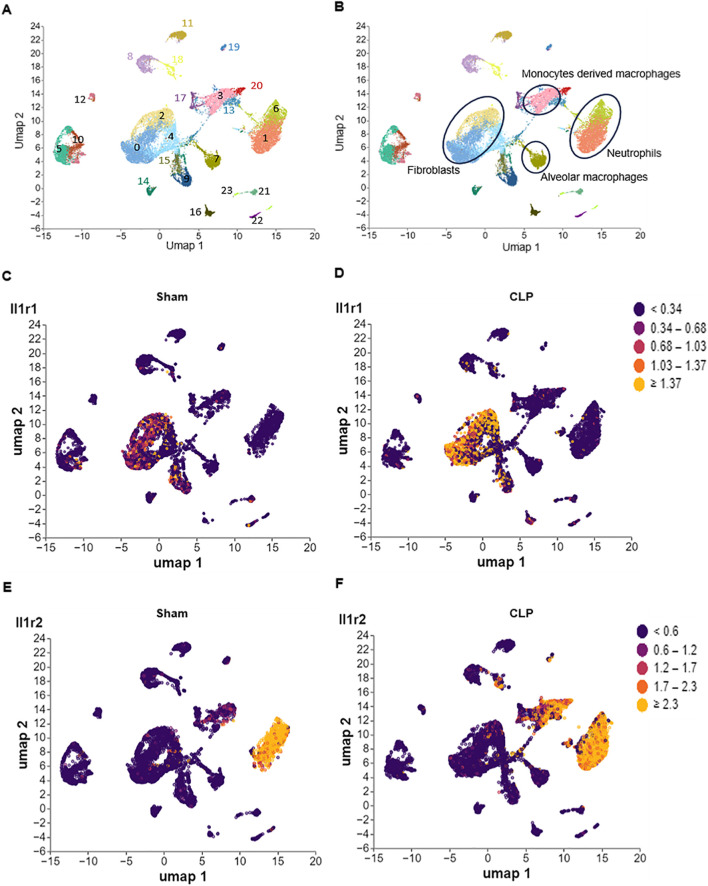
Differential expression of IL-1R1 and IL-1R2 in murine lung during sepsis. Single-cell RNA sequencing (scRNA-seq) analysis of IL-1 receptor expression in lung tissues from sham and CLP induced sepsis mice. **(A)** scRNA-seq data from mouse lung tissues of sham and sepsis mice were analyzed and visualized using uniform manifold approximation and projection (UMAP) plots, with colors in parentheses indicating the identified cell clusters. **(B,C)** UMAP representation of scRNA-seq data from lung cells of sham and septic mice colored according to IL-1R1 expression. **(D–F)** UMAP representation of scRNA-seq data from lung cells of sham and septic mice colored according to IL-1R2 expression.

We downloaded a dataset (GSE207651) from the gene expression omnibus (GEO) database (http://www.ncbi.nlm.nih.gov/geo) (Wang et al., 2022), then analyzed the dataset by Cellecnics platform (https://www.biomage.net/), an open-source platform to analyze single-cell RNA sequencing (scRNA-seq) datasets. The GSE207651 dataset contained gene expression profiling based on mixed single lung cell samples collected for sham specimen, cecum ligation and puncture (CLP)-24 h specimen, CLP-48 h specimen underwent Cellular Indexing of Transcriptomes and Epitopes by Sequencing (CITE-seq). The prepared single cell suspensions were subjected to magnetic-activated cell sorting (MACS) using Miltenyi mouse CD45 magnetic beads according to the manufacturer’s recommendations. The CD45^−^ and CD45^+^ cell suspensions were mixed 1:1 for single-cell sequencing. Filtering of barcodes and quality control of the dataset were finished before upload to GEO database. Different immune cell clusters were annotated based on well-known marker genes and showed in the Supplementary Figure S1. At the end, the IL-1R1/2 expression in different clusters was showed in Figure 1.

### 2.2 Assessment of IL-1R1 and IL-1R2 expression in different cells of murine liver by scRNA-seq data

**FIGURE 2:**
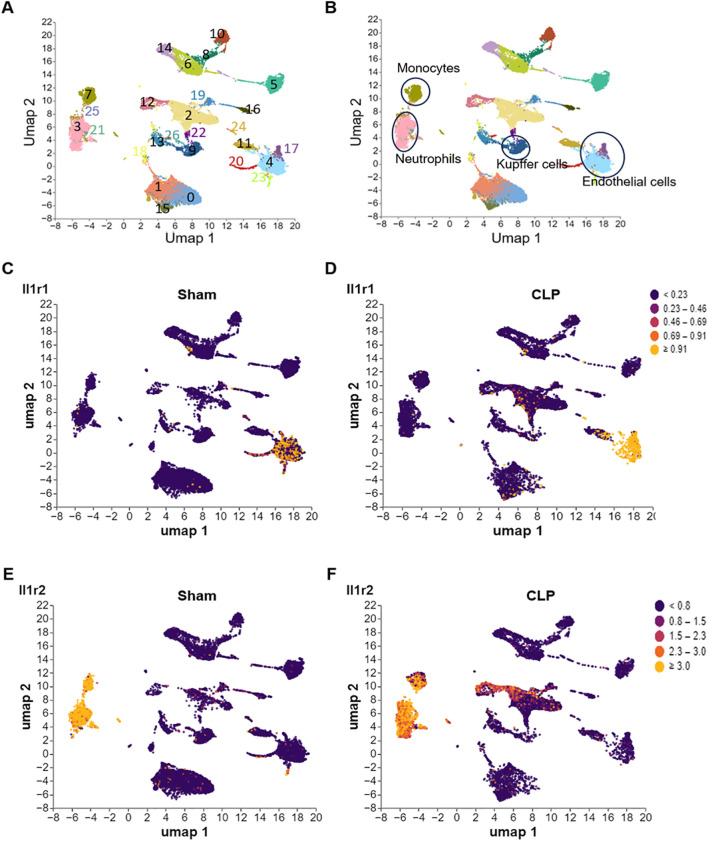
Differential expression of IL-1R1 and IL-1R2 in murine liver during sepsis. scRNA-seq analysis of IL-1 receptor expression in liver tissues from sham and CLP induced sepsis mice. **(A)** scRNA-seq data from mouse liver tissues of sham and septic mice were analyzed and visualized using UMAP plots, with colors in parentheses indicating the identified cell clusters. **(B,C)** UMAP representation of scRNA-seq data from liver cells of sham and septic mice colored according to IL-1R1 expression. **(D–F)** UMAP representation of scRNA-seq data from liver cells of sham and septic mice colored according to IL-1R2 expression.

We downloaded a dataset GSE279167 from the GEO database (Lavarti et al., 2025), then analyzed the dataset by Cellecnics platform. The dataset contained gene expression profiling based on arrays of single liver cell samples were collected for from 3 to 4 mice (Sham or CLP) for single‐cell RNA sequencing. Sequencing was performed with Novaseq 6000 Illumina platform following 10X Genomics guideline. Filtering of barcodes and quality control of the dataset were finished before upload to GEO database. Different immune cell clusters were annotated based on well-known marker genes and showed in the Supplementary Figure S2. At the end, the IL-1R1/2 expression in different clusters was showed in Figure 2.

### 2.3 Assessment of IL-1R1 and IL-1R2 expression in different cells of murine heart by scRNA-seq data

**FIGURE 3:**
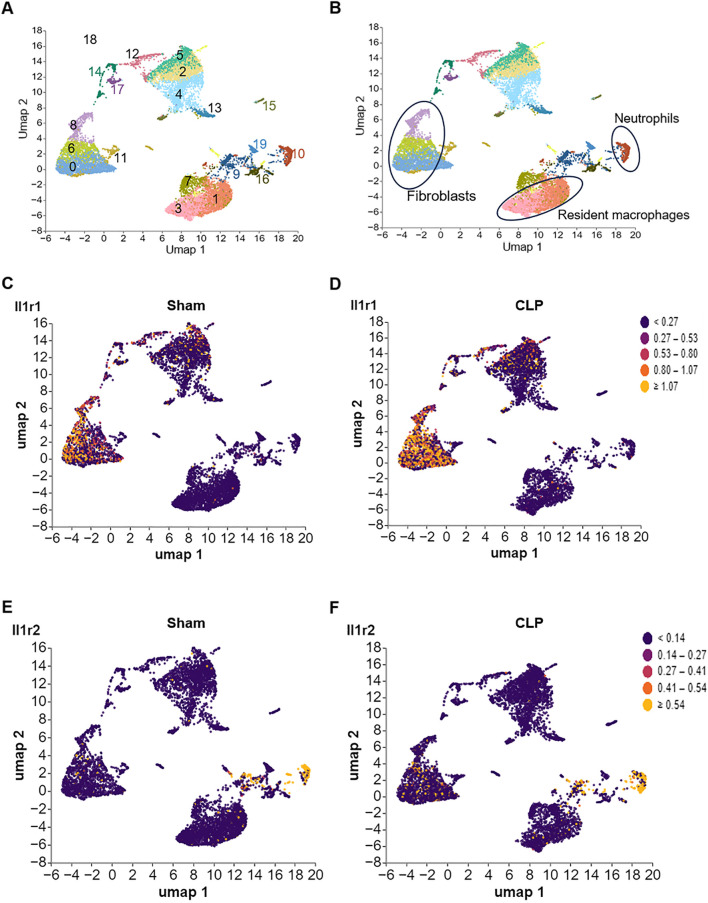
Differential expression of IL-1R1 and IL-1R2 in murine heart tissues during sepsis. scRNA-seq analysis of IL-1 receptor expression in heart tissues from sham and CLP induced sepsis mice. **(A)** scRNA-seq data from mouse heart tissues of sham and sepsis mice were analyzed and visualized using UMAP plots, with colors in parentheses indicating the identified cell clusters. **(B,C)** UMAP representation of scRNA-seq data from heart cells of sham and septic mice colored according to IL-1R1 expression. **(D–F)** UMAP representation of scRNA-seq data from heart cells of sham and septic mice colored according to IL-1R2 expression.

We downloaded a dataset (GSE207363) from the GEO database (Zhao et al., 2024), then analyzed the dataset by Cellecnics platform. The GSE207363 dataset contained gene expression profiling based on mixed single heart cell samples were collected for sham and CLP induced sepsis mice. RNA libraries were prepared with Chromium Single cell 3′ Reagent v3 Kits according to the manufacturer’s protocol. Sequencing was performed with Illumina NovaSeq 6000 platform following 10X Genomics guideline. Filtering of barcodes and quality control of the dataset were finished before upload to GEO database. Different immune cell clusters were annotated based on well-known marker genes and showed in the Supplementary Figure S3. At the end, the IL-1R1/2 expression in different clusters was showed in Figure 3.

### 2.4 Assessment of IL1R1 and IL1R2 expression in different cells of murine small intestine tissue by scRNA-seq data

**FIGURE 4:**
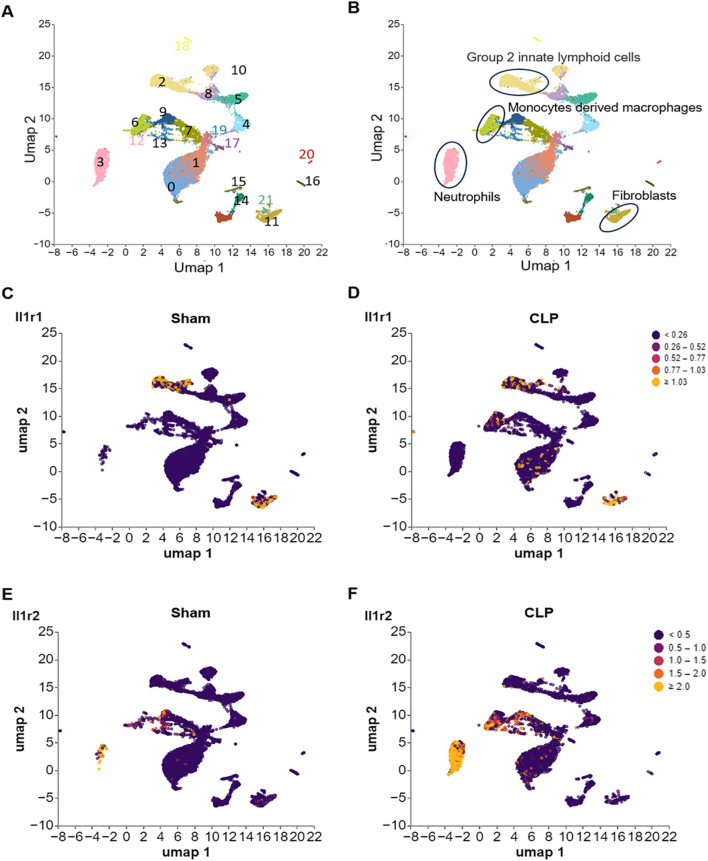
Differential expression of IL-1R1 and IL-1R2 in murine small intestine tissues during sepsis. scRNA-seq analysis of IL-1 receptor expression in small intestine tissues from sham and CLP induced sepsis mice. **(A)** scRNA-seq data from mouse small intestine tissues of sham and sepsis mice were analyzed and visualized using UMAP plots, with colors in parentheses indicating the identified cell clusters. **(B,C)** UMAP representation of scRNA-seq data from small intestine cells of sham and septic mice colored according to IL-1R1 expression. **(D–F)** UMAP representation of scRNA-seq data from small intestine cells of sham and septic mice colored according to IL-1R2 expression.

We downloaded a dataset (GSE266493) from the GEO database (Xie et al., 2024), then analyzed the dataset by Cellecnics platform. The GSE266493 dataset contained gene expression profiling based on arrays of cells from the small intestine tissue of septic and sham-operated C57 mice were isolated fluorescence-activated cell sorting (FACS) for single‐cell RNA sequencing. Prepared single-cell suspensions were loaded on a chip and subjected to a Chromium Single-Cell Instrument (10X Genomics). Filtering of barcodes and quality control of the dataset were finished before upload to GEO database. Clusters were annotated according to the CellMarker database and marker genes of specific cell types reported in research and showed in the Supplementary Figure S4. At the end, the IL-1R1/2 expression in different clusters was showed in Figure 4.

### 2.5 Animals

Male wild-type (WT) C57BL/6J mice, age between 6 and 8 weeks, were purchased from Hunan Slake Jingda Experimental Animals Co. All animals were kept in a 12 h dark/light cycle (25 °C ± 2 °C) under specific pathogen-free (SPF) conditions. The experimental and control animals were housed with unrestricted access to food and water. All animal experiments complied with the National Institutes of Health guidelines for the Care. Animal experiments were conducted in accordance with the Institutional Animal Care and Use Committee of Central South University (NO. 2025-KT065).

### 2.6 Murine model of polymicrobial sepsis

Sepsis was induced in C57BL/6 mice by CLP. In brief, mice were anesthetized with isoflurane and placed in the supine position. A 1 cm midline laparotomy was created in the abdomen of mice to expose the abdominal cavity. The cecum was ligated and punctured with a 22G needle. A consistent and small amount of cecal content was extruded and the cecum was subsequently returned to the peritoneal cavity. Following abdominal closure, resuscitation was facilitated through subcutaneous injection of 500 µL of normal saline. All mice received a single s.c. dose of 0.1 mg/kg buprenorphine after CLP.

### 2.7 Assessment of IL-1R2 expression on murine immune cells in the lung and liver after CLP by flow cytometry

A total of 1 × 10^6^ lung or liver cells were suspended in 100 μL of FACS buffer and stained with FITC- anti-mouse CD64 antibody (Cat. No. 139316, BioLegend, San Diego, CA), APC-Cy7 anti-mouse CD11b antibody (Cat. No. 101226, BioLegend), BV711anti-mouse CD11c (Cat. No. 117349, BioLegend), APC anti-mouse CD45 antibody (Cat. No. 103111, BioLegend), BV421 anti-mouse Ly6G antibody (Cat. No. 127628, BioLegend), FITC- anti-mouse F4/80 antibody (Cat. No. 123108, BioLegend), APC-Cy7 anti-mouse CD45 antibody (Cat. No. 103116, BioLegend), APC anti-mouse Tim4 antibody (Cat. No. 130022, BioLegend), and PE anti-mouse IL-1R2 antibody (Cat. No. 554450, BD Biosciences, San Jose, CA) for 30 min at room temperature. PE rat IgG2 antibody (Cat. No. 553930, BD Biosciences) was used as an isotype Ab. Unstained cells were used to establish control voltage settings and single-color compensation was based with UltraComp eBeads (Thermo-Fisher, Waltham, MA). Acquisition was performed on 20,000 events using a BD FACSymphony™ A1 flow cytometer (BD Biosciences, San Jose, CA) and data were analyzed with FlowJo software (Tree Star, Ashland, OR). We gated the cells in FSC and SSC to exclude debris or dead cells, considering that apoptotic cells/debris are located near the X- and Y-axis. We also excluded doublets using FSC-A/FSC-H gating.

### 2.8 Assessment of IL-1R2 expression on murine blood cells after CLP by flow cytometry

A total of 100 µL blood cells were stained with APC anti-mouse CD11b antibody (Cat. No. 101212, BioLegend), FITC- anti-mouse Ly6C antibody (Cat. No. 128006, BioLegend), APC-Cy7 anti-mouse Ly6G antibody (Cat. No. 103116, BioLegend), and PE anti-mouse IL-1R2 antibody (Cat. No. 554450, BD Biosciences, San Jose, CA) for 30 min at room temperature. PE rat IgG2 antibody (Cat. No. 553930, BD Biosciences) was used as an isotype Ab. The cells were analyzed using a BD FACSymphony™ A1 flow cytometer (BD Biosciences, San Jose, CA) and data were analyzed with FlowJo software (Tree Star, Ashland, OR).

### 2.9 Statistical analysis

All statistical analyses were performed and the figures were prepared with GraphPad Prism version 8.0 software (GraphPad Software, La Jolla, CA). Comparisons between two groups were performed with a two-tailed Student’s t-test (parametric). Comparisons between multiple groups were analyzed using a one-way analysis of variance (ANOVA), followed by Tukey’s multiple comparison test. The statistical significance was set at p < 0.05.

## 3 Results

### 3.1 The expression of IL-1R1 and IL-1R2 in the lung tissue of septic mice

To investigate the expression patterns of IL-1R in the lung tissues, we analyzed published scRNA-seq data of the lung from health and septic mice. We found that IL-1R1 was predominantly expressed in fibroblasts (clusters 0 and 2), identified by high expression of *Pdgfra* and *Col1a1* (Figures 1A–D; Supplementary Figures S1A,B). Compared with Sham mice, the expression of IL-1R1 was increased in these cells of septic mice (Figures 1C,D). In contrast, IL-1R2 primarily enriched in recruited myeloid subsets, including neutrophils (cluster 1 and 6, marked by co-expressed by *S100a8* and *S100a9*) and monocyte-derived macrophages (cluster 3, marked by co-expressed by *Adgre1* and *Cd14*) (Figures 1A,B,E,F; Supplementary Figures S1C,D). Compared with Sham mice, the expression of IL-1R2 was significantly upregulated in neutrophils and monocyte-derived macrophages of septic mice (Figures 1E,F). In addition, IL-1R2 expression is minimal in resident alveolar macrophages (cluster 7, marked by co-expressed by *Adgre1*, *Itgax* and *Siglecf*) (Figures 1A,B,E,F; Supplementary Figures S1E,G,H). Despite the marked upregulation of IL-1R2 on recruited myeloid cells, we observed a striking lack of IL-1R2 upregulation in resident alveolar macrophages. These findings establish the distinct cellular landscapes of IL-1R1 and IL-1R2 expression in the lung tissue of septic mice.

### 3.2 The expression of IL-1R1 and IL-1R2 in the liver tissue of septic mice

To explore the expression of IL-1 receptors in the liver, we analyzed published scRNA-seq data of the liver from health and septic mice. We found that IL-1R1 was predominantly expressed in the sinusoidal endothelial cells (clusters 4 and 17) characterized by co-expressed with *Lyve1* and *Col1a1* (Figures 2A–D; Supplementary Figures S2A,B), and its expression was increased in these cells of septic mice (Supplementary Figure S2). In contrast, IL-1R2 expression was largely restricted to neutrophils (cluster 3, *S100a8* and *Ly6g*) and monocytes derived macrophages (cluster 7, Adgre1 and *Cd14*) of liver tissues (Figures 2A,B,E,F; Supplementary Figures S2C–F). Compared with Sham mice, the expression of IL-1R2 was significantly upregulated in these cells of septic mice (Figures 2E,F). In addition, IL-1R2 expression is minimal in cluster 9 (Figures 2A,B,E,F), which co-expressed *Adgre1*, *Clec4f* and *Tim4* and identified as resident macrophages, namely Kupffer cells in the liver (Supplementary Figures S2E,G,H), and this expression did not increase in response to sepsis. These findings establish the distinct cellular landscapes of IL-1R1 and IL-1R2 expression in the liver tissue.

### 3.3 The expression of IL-1R1 and IL-1R2 in the heart of septic mice

To establish the baseline expression patterns of IL-1R, we analyzed published scRNA-seq data of the heart from health and septic mice. We found that IL-1R1 was predominantly expressed in cell cluster 0, 6 and 8 (Figures 3A–D). Based on the co-expression of established markers *Col1a1* and *Pdgfr2*, we identified these three clusters as heart fibroblasts (Supplementary Figures S3A,B). Compared with Sham mice, the expression of IL-1R1 was slightly increased in these cells. In contrast, IL-1R2 expression was largely restricted to cluster 10 (Figures 3A,C), which was identified as neutrophils based on the expression of markers S1008a and S1009a (Supplementary Figures S3C,D). Compared with Sham mice, the expression of IL-1R2 was slightly upregulated in these cells (Figures 3E,F). In addition, IL-1R2 expression is minimal in cluster 1 and 3 under health and sepsis condition (Figures 3A,B,E,F), which co-expressed with *Fcgr1*and *Tim4 but lack of Cxcr2* (Supplementary Figures S3E–G), identified as resident tissue macrophages in the heart. These findings establish the distinct cellular landscapes of IL-1R1 and IL-1R2 expression in the heart tissue.

### 3.4 The expression of IL-1R1 and IL-1R2 in the small intestine tissue of septic mice

Analysis of published scRNA-seq data from healthy and septic murine small intestine tissues revealed distinct, cell-type-specific expression profiles for the IL-1R (Figure 4). IL-1R1 expression was primarily observed in cluster 2, identified as group 2 innate lymphoid cells (ILC2s) based on the marker *Gata3* (Figures 4A–D; Supplementary Figure S4A), and cluster 11, identified as fibroblasts via the marker *Col1a1* (Figures 4A–D; Supplementary Figure S4B). Notably, IL-1R1 expression in these populations showed no significant change between Sham and septic mice (Figures 4C,D). In contrast, IL-1R2 expression was largely restricted to immune cells (Figures 3A,B,E,F): cluster 3 identified as neutrophils (marked by S1008a and S1009a; Supplementary Figures S4C,D) and cluster 6 identified as monocyte-derived macrophages (marked by Fcgr1 and Cd14; Supplementary Figures S4E,F). Critically, IL-1R2 expression in both these cell types was significantly upregulated in septic animals compared to controls (Figures 4E,F). Conversely, IL-1R2 expression was minimal across both healthy and septic conditions in cluster 9, which co-expressed *Fcgr1, Cx3cr1*, and *C1qb*, identifying it as the population of resident tissue macrophages (Supplementary Figures S4E,G,H). These data establish a compartmentalized expression landscape for IL-1R1 and IL-1R2 in the small intestine, highlighting differential regulatory mechanisms during sepsis.

### 3.5 Flow cytometry validation of IL-1R2 expression patterns in the lung and liver

**FIGURE 5:**
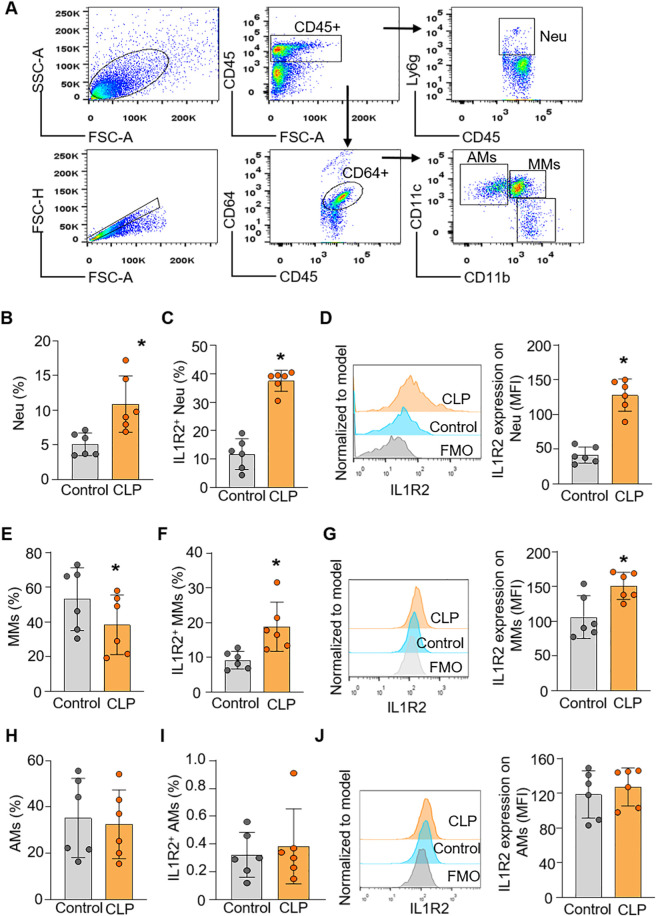
Flow cytometric analysis of IL-1R2 protein expression in murine lung during health and sepsis. C57BL/6 mice were subjected to sham operation or CLP-induced sepsis. After 24 h of CLP, the lung tissues were collected for flow cytometry. **(A)** Representative flow cytometry gating strategy used to identify neutrophils, and macrophages in the lung. **(B)** The percentage of neutrophils in the lung tissues of mice. **(C,D)** The frequencies of IL-1R2 expression on neutrophils and histogram showing mean immunofluorescence intensity (MFI) of IL-1R2 expressing are shown. **(E)** The percentages of alveolar macrophages (AMs) in the lung tissues of mice. **(F,G)** The frequencies of IL-1R2 expression on AMs and histogram showing mean MFI of IL-1R2 expressing are shown. **(H)** The percentages of monocytes derived macrophages (MMs) in the lung tissues of mice. **(I,J)** The frequencies of IL-1R2 expression on MMs and histogram showing the MFI of IL-1R2 expressing is shown. Data are expressed as means ± SE (Control group, *n* = 6 mice; CLP group, *n* = 6 mice). The groups were compared by a two-tailed Student’s t-test. (*p < 0.05 vs. Control group).

**FIGURE 6:**
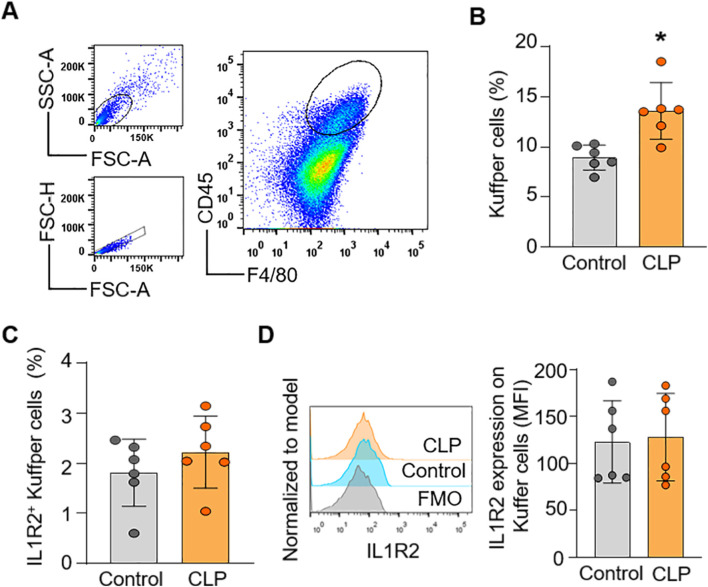
Flow cytometric analysis of IL-1R2 protein expression in murine liver during health and sepsis. C57BL/6 mice were subjected to sham operation or CLP-induced sepsis. After 24 h of CLP, the livers were collected for flow cytometry. **(A)** Representative flow cytometry gating strategy used to identify macrophages (Kuffper cells) in the liver. **(B)** Histograms showing the percentage of Kuffper cells in the liver of mice. **(C,D)** Histograms showing the percentage of IL-1R2 expression on Kuffper cells and histogram showing MFI of IL-1R2 expressing cells are shown. The figures represent the results of two times experimental data together. Data are expressed as means ± SE (Control group, *n* = 6 mice; CLP group, *n* = 8 mice). The groups were compared by a two-tailed Student’s t-test. (*p < 0.05 vs. Control group).

**FIGURE 7:**
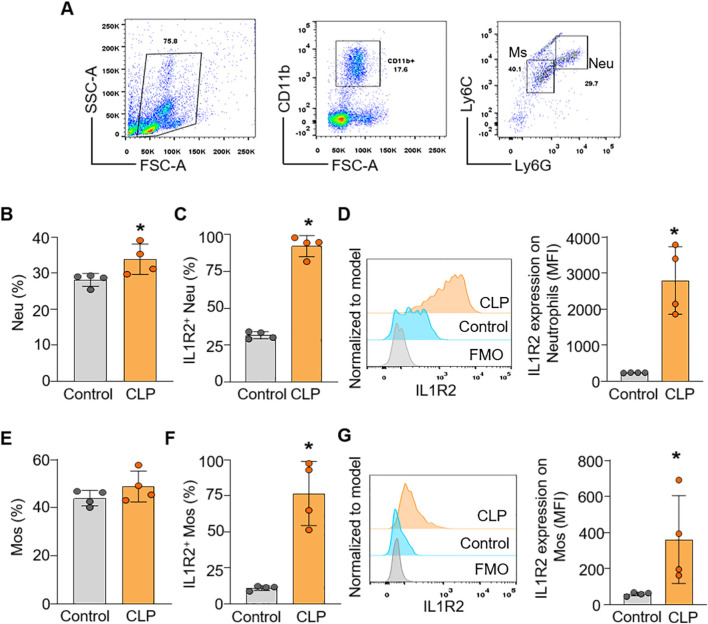
Flow cytometric analysis of IL-1R2 protein expression in murine blood during health and sepsis. C57BL/6 mice were subjected to sham operation or CLP-induced sepsis. After 24 h of CLP, the blood was collected for flow cytometry. **(A)** Representative flow cytometry gating strategy used to identify neutrophils, and monocytes in the blood. **(B)** The percentages of neutrophils (Neu) in the blood of mice. **(C,D)** The frequencies of IL-1R2 expression on neutrophils and histogram showing MFI of IL-1R2 expressing are shown. **(E)** The percentages of monocytes (Mos) in the blood of mice. **(F,G)** The frequencies of IL-1R2 expression on Mos and histogram showing mean MFI of IL-1R2 expressing are shown. Data are expressed as means ± SE (*n* = 4 mice/group). The figures represent the results of one experimental data and the experiment repeated three times. The groups were compared by a two-tailed Student’s t-test. (*p < 0.05 vs. Control group).

To confirm the findings from scRNA-seq data at the protein level of IL-1R2, we performed flow cytometry analysis on lung and liver tissues from healthy and septic mice. Consistent with the scRNA-seq data, flow cytometry confirmed that IL-1R2 was significantly upregulated on neutrophils and monocytes derived macrophages in the lung during sepsis (Figures 5A–G). Despite the marked upregulation of IL-1R2 on recruited myeloid cells, we observed that alveolar macrophages in the lung tissue maintained low levels of IL1R2 expression even during sepsis, comparable to their expression levels in healthy conditions (Figures 5H–J). Flow cytometry also confirmed that the resident tissue macrophages, kupffer cells, in the liver maintained low levels of IL-1R2 expression even during sepsis, comparable to their expression levels in sham mice (Figures 6A–D). In addition, flow cytometry confirmed that IL-1R2 protein were significantly upregulated on neutrophils in the blood during sepsis (Figures 7A–D). And IL-1R2 protein was significantly increased on monocytes in the blood during sepsis (Figures 7E–G). These flow cytometry results corroborate the scRNA-seq findings, demonstrating a consistent and cell-type-specific regulation of IL-1R protein expression during sepsis.

## 4 Discussion

Our study provides a comprehensive analysis of IL-1R1 and IL-1R2 expression across different cell populations and organs during sepsis by analyzing published sc-RNAseq data and flow cytometry. We identified an interesting finding: while IL-1R1 is primarily expressed on non-immune structural cells, IL-1R2 is selectively upregulated on infiltrating neutrophils and monocyte-derived macrophages, but not on resident tissue macrophages. This distinction raises important questions regarding the differential roles of IL-1R1 and IL-1R2 in innate immunity and inflammation during sepsis.

IL-1R1 functions as the primary signaling receptor for both IL-1α and IL-1β. Upon binding to these ligands, IL-1R1 recruits the accessory protein IL-1RAcP, leading to the formation of a signaling complex that subsequently activates downstream intracellular pathways, including the NF-κB and mitogen-activated protein kinase (MAPK) pathways. IL-1R1 has been found in all cells of the innate immune system, encompassing macrophages, neutrophils, in addition to selective T cell populations within the adaptive immune system (Ben-Sasson et al., 2013; Sims and Smith, 2010). However, the expression of IL-1R1 in peripheral non-immune cells is receiving increasing attention. Our study demonstrated that, under both healthy and septic conditions, IL-1R1 is selectively highly expressed on non-immune structural cells, such as lung fibroblasts, liver sinusoidal endothelial cells, heart fibroblasts and intestine fibroblasts. This finding is consistent with previous research demonstrating that IL-1R1 is expressed on endothelial cells in the brain tissues, but not expressed on microglia and astrocytes (Liu et al., 2015). They also found that fibroblastic reticular cells and endothelial cells, rather than leukocytes, as the cells exhibiting the highest IL-1R1 in colon, muscle, and many immune organs (Song et al., 2018). These findings indicate that structural non-immune cells, rather than the mobile immune cells, are the preeminent expressors of IL-1R1, suggesting that these cells represent the initial and most critical responders to systemic or local IL-1 signaling. Notably, our study also identifies a unique expression pattern of IL-1R1 in ILC2 in the intestine tissues under both healthy and sepsis condition. This observation suggests a potential immunomodulatory role for IL-1R1 in regulating ILC2 function, particularly in the context of mucosal immunity and inflammation. However, further investigation is needed to elucidate the precise role by which IL-1R1 influences innate lymphoid cell in sepsis.

In contrast, IL-1R2, as a decoy receptor, binds IL-1α and IL-1β with high affinity, preventing their interaction with the signaling receptor IL-1R1 and thereby reducing downstream pro-inflammatory signaling, including the production of other cytokines like TNF-α and IL-6 (Colotta et al., 1993; Mantovani et al., 2001; Sims et al., 1993). In current study, we observed that IL-1R2 is selectively upregulated on infiltrating neutrophils and monocyte-derived macrophages across multiple tissues in septic mice. Our published data has also demonstrated that IL1R2 is primarily expressed by monocytes and neutrophils in the blood of septic patients (Tan et al., 2025). The significant upregulation of IL-1R2 on recruited neutrophils and monocyte-derived macrophages likely represents a crucial mechanism for balancing the systemic inflammatory response. This is consistent with previous studies showing that IL-1R2 is protective in various inflammatory disease models, including models of sepsis, and sterile inflammation (Zhang et al., 2024; Tan et al., 2025; Martin et al., 2017; Shimizu et al., 2015). Researchers utilized mice with monocyte’s conditional IL-1R2 deletion demonstrated that IL-1R2 acts as a negative regulator of monocyte recruitment during inflammation by sequestering IL1-β and reducing CCL2-mediated monocyte trafficking. In mouse models of peritonitis and neuro-inflammation, IL-1R2 deficiency led to increased monocyte infiltration and exacerbated disease severity (Cros and Segura, 2025). Furthermore, one recent study revealed that IL-1R2 is selectively upregulated on monocytes and is associated with their differentiation into a unique circulating monocytic cell population (Supino et al., 2025). This IL-1R2^+^ Monocytes subset exhibits hallmarks of immune dysfunction, including low expression of HLA-DR and high levels of macrophage markers (MS4A4A, CD63) and immune checkpoints (Supino et al., 2025). Importantly, the expression of membrane-associated IL-1R2 and MS4A4A in these circulating cells correlates with clinical severity markers (e.g., SOFA score, creatinine, cytokine storm), reflecting the infection’s poor prognosis (Supino et al., 2025). Therefore, the increased IL-1R2 expression on newly recruited neutrophils and monocyte-derived macrophages can be interpreted as a negative feedback mechanism triggered by high IL-1 levels, which not only scavenges inflammatory ligand to limit acute damage but also marks a distinct population of monocytes/macrophages associated with the overall severity and immune dysfunction in sepsis.

A particularly significant finding in this study is the minimal expression of IL-1R2 on resident innate immune cell, like tissue resident macrophages and innate lymphoid cells. These cells play vital roles in maintaining tissue homeostasis, clearing pathogens through phagocytosis, and orchestrating local immune responses by producing pro- and anti-inflammatory cytokines (Davies et al., 2013; Wynn et al., 2013; Epelman et al., 2014). IL-1 signaling enhances the phagocytic capacity of macrophages, promoting the clearance of bacteria and apoptotic cells (Dinarello, 2011; Kono and Rock, 2008; Blander and Medzhitov, 2004), processes essential for both pathogen control and the resolution of inflammation. These resident macrophages maintain low IL-1R2 expression to preserve sensitivity to IL-1 may be crucial for their local functions within the tissue microenvironment. However, a study identified a reduced expression of IL-1R1 mRNA and an elevated expression of IL-1R2 mRNA in microglia, the resident macrophages of the mouse brain (Pinteaux et al., 2002). The differential expression of IL-1R2 likely reflects the distinct origins, transcriptional programs, and epigenetic landscapes of resident versus recruited myeloid cells (Lavin et al., 2014; Gautier et al., 2012; Gosselin et al., 2014). Resident macrophages, such as Kupffer cells and microglia, originate from fetal precursors and maintain themselves through local proliferation, whereas intestinal macrophages and certain skin macrophages are replenished throughout life from circulating bone marrow–derived monocytes. They possess unique transcriptional profiles compared to monocyte-derived macrophages that are recruited from the circulation in response to inflammatory signals (Yona et al., 2013; Schulz et al., 2012). These differences in developmental origin and transcriptional regulation may render resident macrophages less responsive to the systemic inflammatory signals that drive IL-1R2 upregulation in recruited cells. The specific molecular mechanisms underlying this differential regulation warrant further investigation.

Several limitations should be acknowledged in this study. First, the use of four different databases with varying sampling time frames may introduce heterogeneity that could influence the interpretation of the results. While this approach allowed for broader validation, differences in sample collection and processing across datasets could impact consistency. Second, our experimental sepsis model primarily utilized young, healthy male mice. Although widely accepted in preclinical research, this model does not fully capture the clinical reality of sepsis, which predominantly affects older individuals with comorbidities. While replication of our study in aged mice is beyond the current scope, future research should consider incorporating more clinically relevant models to improve translational value.

**FIGURE 8:**
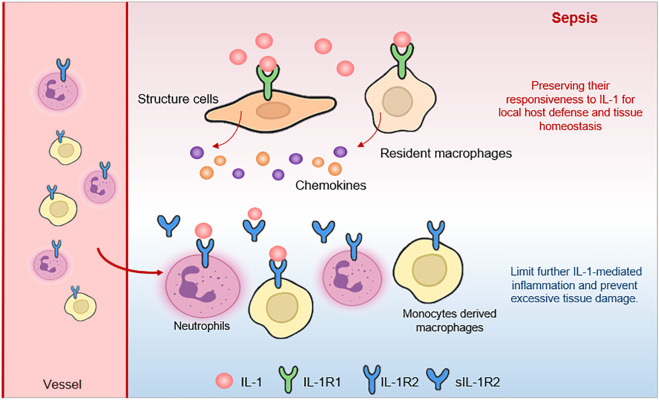
The schematic illustrates how different cell populations regulate IL-1 signaling in the septic state. Structural cells (fibroblasts and endothelial cells) maintain expression of the signaling receptor IL-1R1 (green), allowing them to respond to IL-1 (red). Resident macrophages also remain sensitive to IL-1 through IL-1R1, supporting local defense and clearance functions. In contrast, recruited immune cells (such as neutrophils and monocyte-derived macrophages) upregulate the decoy receptor IL-1R2 (blue), which binds excess IL-1 without initiating signaling. This receptor acts as a molecular to buffer against systemic hyperinflammation, while local tissue macrophages continue to respond to IL-1 signals.

In conclusion, our study reveals a cell-type-specific and organ-consistent regulation of IL-1Rs during sepsis. We demonstrate that recruited neutrophils and monocyte-derived macrophages significantly upregulate IL-1R2, likely as a mechanism to balance systemic inflammation, while resident tissue macrophages (alveolar macrophages and Kupffer cells) maintain low IL-1R2 expression, preserving their responsiveness to IL-1 for local host defense and tissue homeostasis (Figure 8). This differential regulation highlights a sophisticated mechanism by which the host immune system balances the need for systemic control of inflammation with the requirement for local immune competence.

## Data Availability

The datasets presented in this study can be found in online repositories. The names of the repository/repositories and accession number(s) can be found below: https://www.ncbi.nlm.nih.gov/, GSE266493 https://www.ncbi.nlm.nih.gov/, GSE207363 https://www.ncbi.nlm.nih.gov/, GSE279167 https://www.ncbi.nlm.nih.gov/, GSE207651.
