# Supplementary material for: Decoding IL-1 receptor 1 and 2 expression profiles across organs in sepsis
Source: Front Cell Dev Biol. 2025 Nov 10;13:1675870. doi: 10.3389/fcell.2025.1675870 (PMC12640944; doi:10.3389/fcell.2025.1675870)
Supplement: Supplementary file 1 [file DataSheet1.docx]

**Supplementary figure legends and figures**


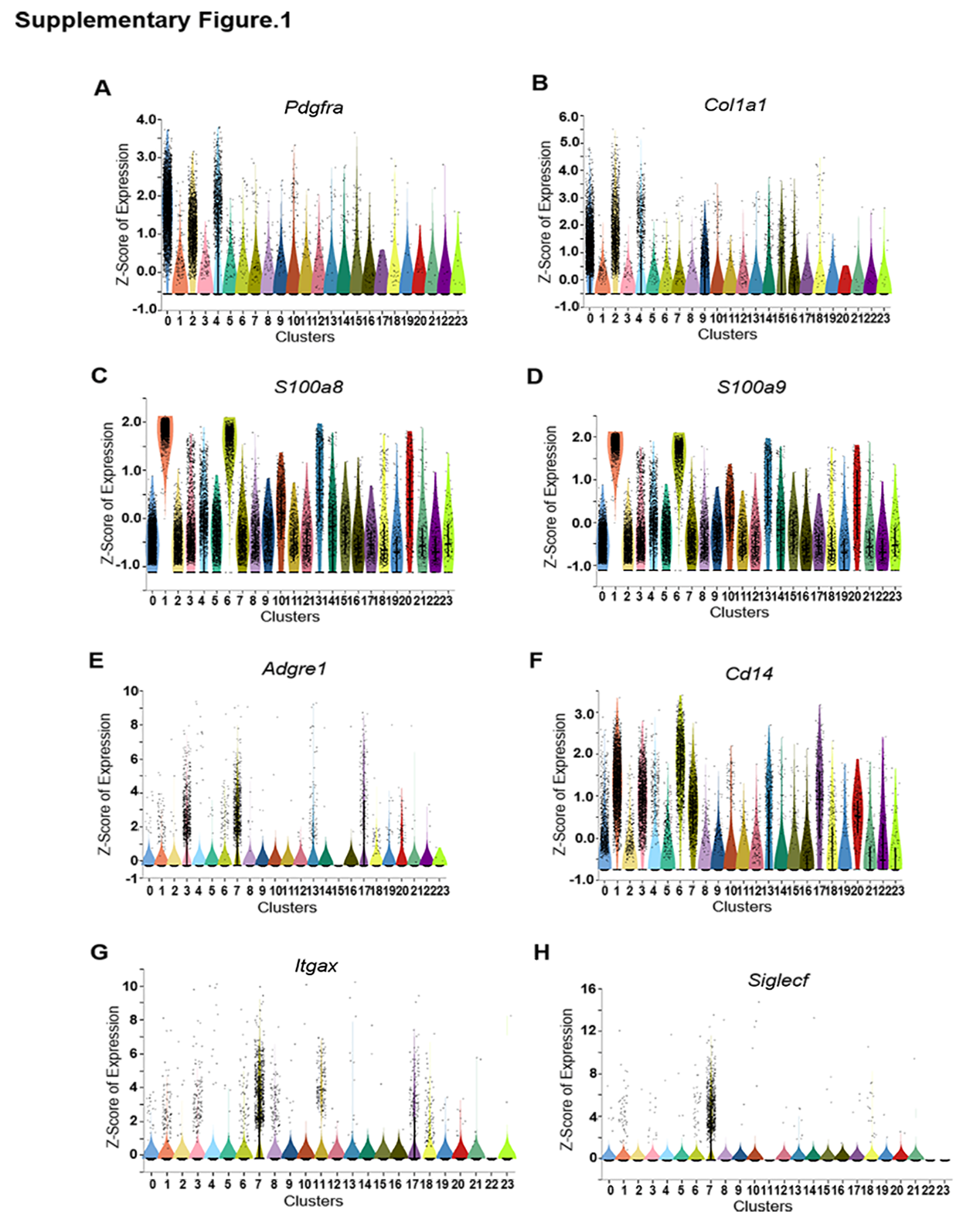


**Supplementary figure.1 The cell markers expression of cell clusters in murine lung tissues.** (A-B) Annotation of lung fibroblast was performed manually using expression of *Pdgfra* (A) and *Col1a1* (B). (C-D) Annotation of neutrophils was performed manually using expression of *S100a8* (C) and *S100a9* (D). (E-F) Annotation of monocytes derived macrophage was performed manually using expression of *Adgre1* (E) and *Cd14* (F). (G-H) Annotation of alveolar macrophage was performed manually using expression of *Itgax* (G) and *Siglecf* (H).

**
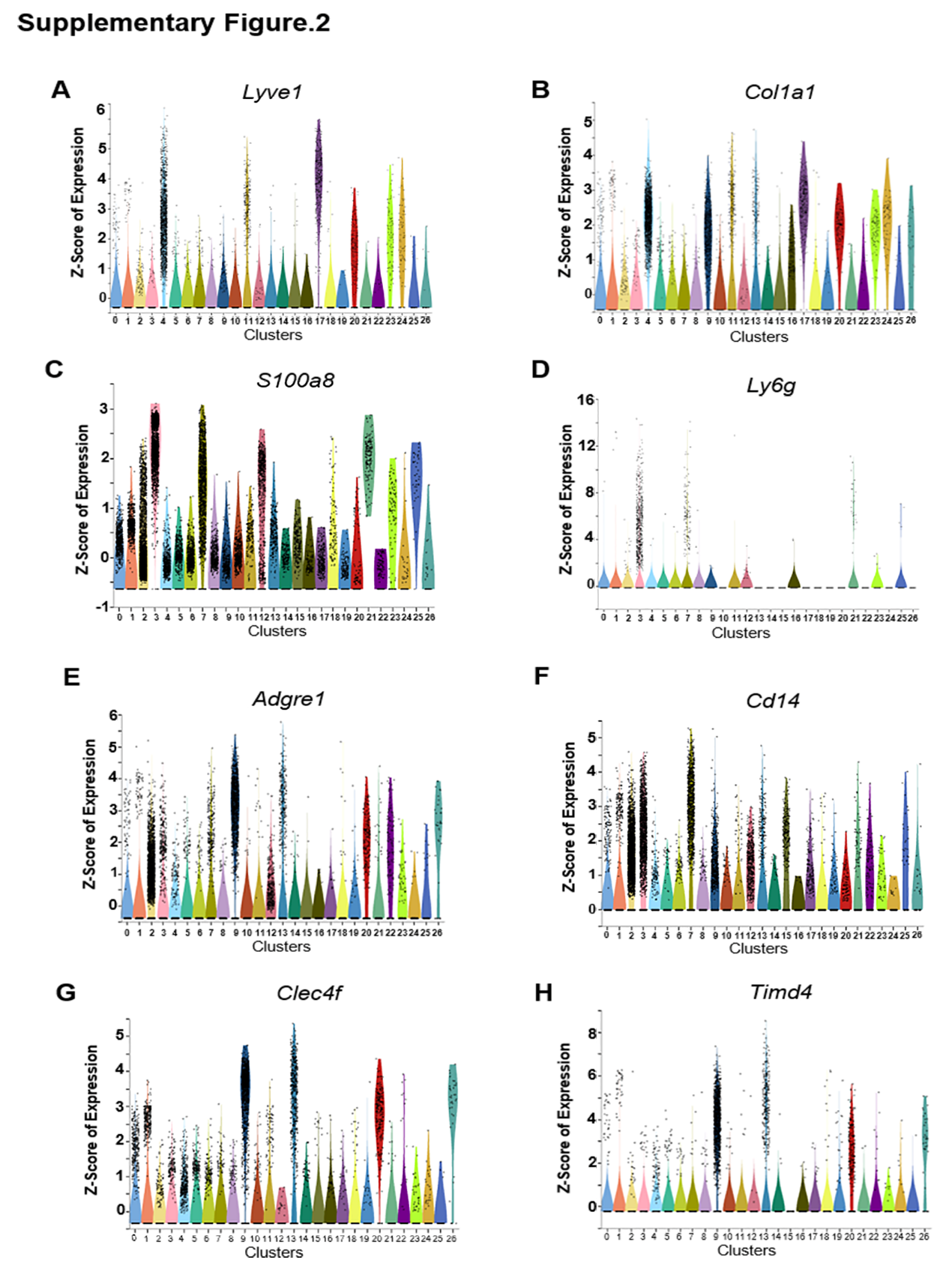
Supplementary figure.2 The cell markers expression of cell clusters in murine liver tissues.** (A-B) Annotation of liver sinusoidal endothelial cells was performed manually using expression of *Lyve1* (A) and *Col1a1* (B). (C-D) Annotation of neutrophils was performed manually using expression of *S100a8* (C) and *S100a9* (D). (E-F) Annotation of monocytes derived macrophage was performed manually using expression of *Adgre1* (E) and *Cd14* (F) (G-H) Annotation of Kupffer cell was performed manually using expression of *Clec4f* (G) and *Timd4* (H).

**
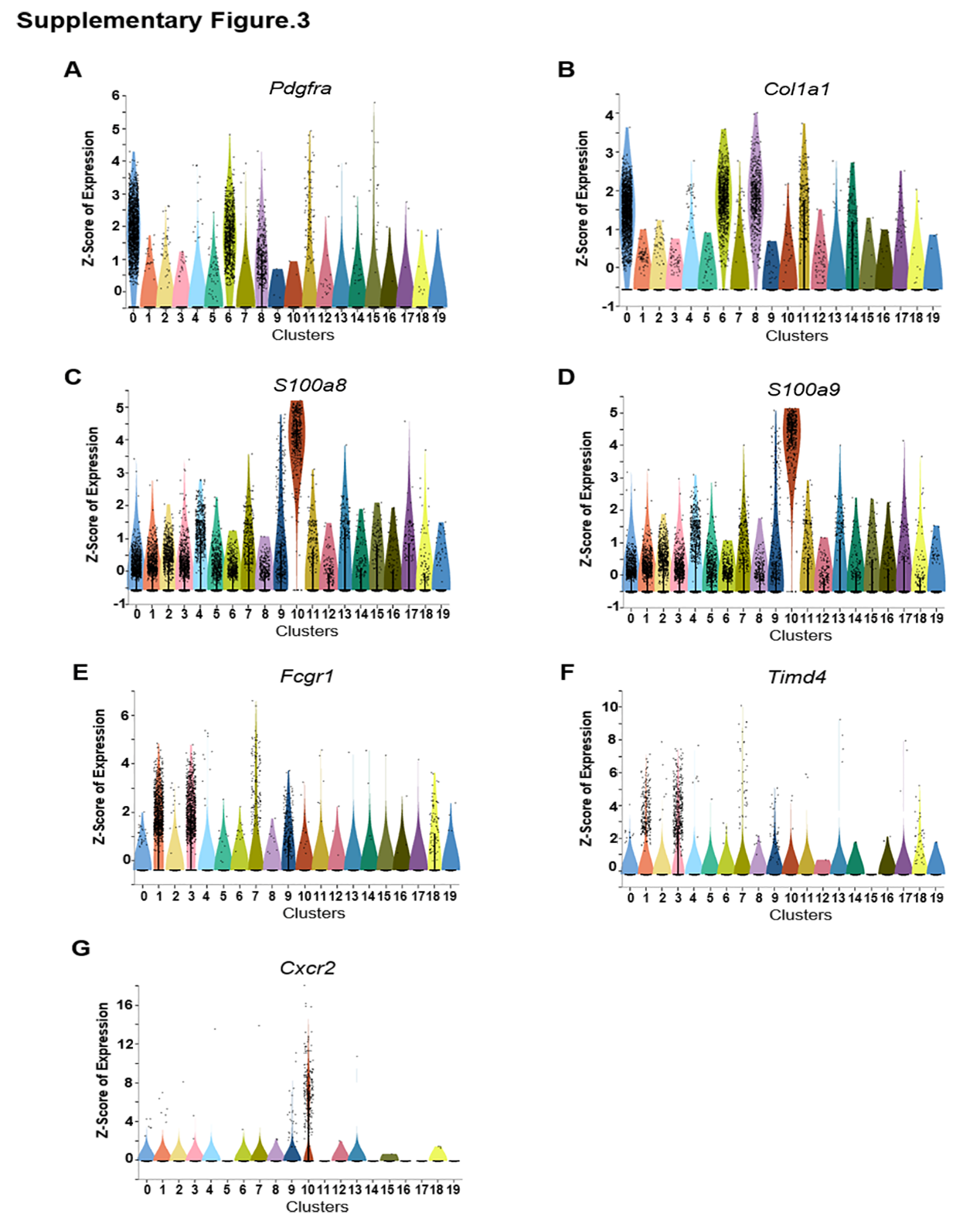
Supplementary figure.3 The cell markers expression of cell clusters in murine heart tissues.** (A-B) Annotation of heart fibroblast was performed manually using expression of *Pdgfra* (A) and *Col1a1* (B). (C-D) Annotation of neutrophils was performed manually using expression of *S100a8* (C) and *S100a9* (D). (E-G) Annotation of resident macrophage was performed manually using expression of *Fcgr1*(E), *Clec4f* (F) and *Timd4* (G).

**
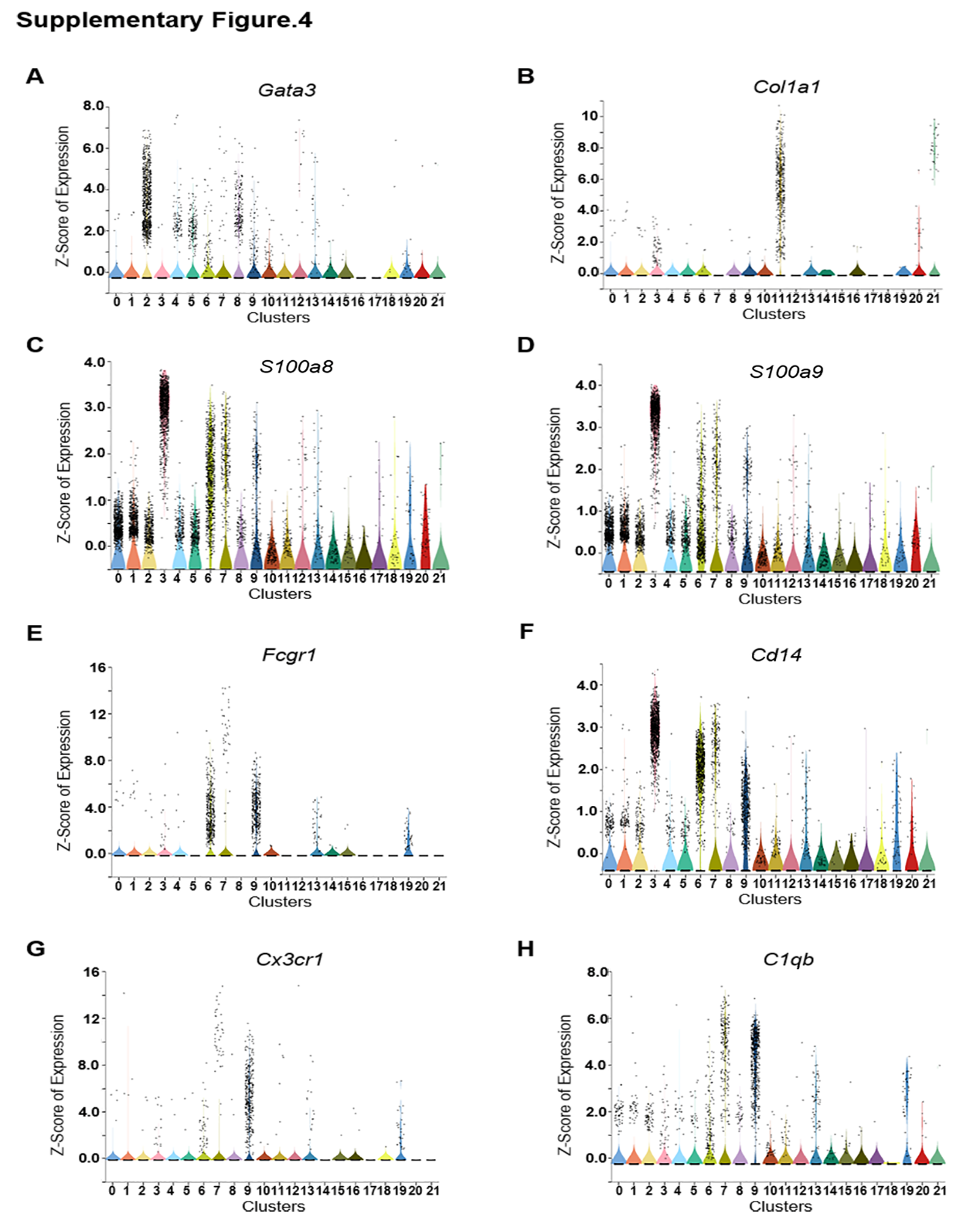
**

**Supplementary figure.4 The cell markers expression of cell clusters in murine small intestine tissues.** (A) Annotation of intestine fibroblast was performed manually using expression of *Col1a1*. (B) Annotation of intestine group2 innate lymphoid cell was performed manually using expression of *Gata3*. (C-D) Annotation of neutrophils was performed manually using expression of *S100a8* (C) and *S100a9* (D). (E-F) Annotation of monocytes derived macrophage was performed manually using expression of *Adgre1* (E) and *Cd14* (F). (G-H) Annotation of resident macrophage was performed manually using expression of *Cx3cr1*(G) and *C1qbf* (H).
